# Neural function of *Bmal1*: an overview

**DOI:** 10.1186/s13578-022-00947-8

**Published:** 2023-01-02

**Authors:** Yuanjia Zheng, Lingyun Pan, Feixue Wang, Jinglan Yan, Taiyi Wang, Yucen Xia, Lin Yao, Kelin Deng, Yuqi Zheng, Xiaoye Xia, Zhikai Su, Hongjie Chen, Jie Lin, Zhenwei Ding, Kaitong Zhang, Meng Zhang, Yongjun Chen

**Affiliations:** 1 Research Institute of Acupuncture and Moxibustion, Shandong University of Traditional Chinese Medicine, Jinan, China; 2 South China Research Center for Acupuncture and Moxibustion, Medical College of Acu-Moxi and Rehabilitation, Guangzhou University of Chinese Medicine, Guangzhou, China; 3 Center for Brain Science and Brain-Inspired Intelligence, Guangdong-Hong Kong-Macao Greater Bay Area, Guangzhou, China; 4 The Second Clinical College, Guangzhou University of Chinese Medicine, Guangzhou, Guangdong China

**Keywords:** *Bmal1*, Pleiotropy, Neurobiology, Neural function, Mental disorder, Biological clock

## Abstract

*Bmal1* (Brain and muscle arnt-like, or Arntl) is a bHLH/PAS domain transcription factor central to the transcription/translation feedback loop of the biologic clock. Although *Bmal1* is well-established as a major regulator of circadian rhythm, a growing number of studies in recent years have shown that dysfunction of *Bmal1* underlies a variety of psychiatric, neurodegenerative-like, and endocrine metabolism-related disorders, as well as potential oncogenic roles. In this review, we systematically summarized *Bmal1* expression in different brain regions, its neurological functions related or not to circadian rhythm and biological clock, and pathological phenotypes arising from *Bmal1* knockout. This review also discusses oscillation and rhythmicity, especially in the suprachiasmatic nucleus, and provides perspective on future progress in *Bmal1* research.

## Introduction

*Bmal1* (Brain and muscle arnt-like), also known as *Arntl*, is a bHLH/PAS domain transcription factor that serves as a core factor in the transcription/translation feedback loop (TTFL) of the biological clock. *Bmal1* forms a heterodimer with the protein product encoded by the *Clock* gene. This heterodimer in turn binds genes with E-box elements such as *Per 1, Per 2, Per 3, Cry 1,* and *Cry 2* to activate their transcription. PER and CRY proteins can inhibit CLOCK/BMAL1 heterodimer activity [1, 2], which leads to formation of a negative feedback loop. Recent and ongoing advances in gene targeting technology have enabled closer study of several pathological *Bmal1* deletion phenotypes. These studies collectively support that *Bmal1* deletion or conditional knockdown/knockout can cause circadian rhythm-related disorders, as well as other disease phenotypes that strikingly resemble psychiatric disorders (e.g., depression, schizophrenia, etc.) and neurodegeneration (e.g., Parkinson's syndrome, etc.) [3–7]. In addition, conditional knockdown/knockout of *Bmal1* has also been linked with behavioral abnormalities that occur even while maintaining a normal circadian rhythm [8–10]. However, the mechanisms by which defects in this gene can lead to these neurological diseases have remained unclear, suggesting an incomplete understanding of the genetic basis of the biological clock. Thus, considerable research attention has focused on identifying previously unrecognized functions of the biological clock genes such as *Bmal1*.

Pleiotropy refers to the formation of multiple traits conferred or influenced by a single gene, and thus involves a multiple physiological and can thus simultaneously affect a variety of physiological systems. As a core transcription factor in the TTFL, *Bmal1* participates in maintaining the molecular biological clock of cells and can also mediate the development of a variety of diseases. In this paper, we systematically review studies investigating *Bmal1* expression in the brain, the neurological function(s) of *Bmal1*, and pathological phenotypes arising from *Bmal1* deficiency to comprehensively understand its effects*.*

## Overview of *Bmal1*

Genetic data suggest that *Bmal1* is an important component of the mammalian circadian pacemaker [11, 12]. In mammals, the biological clock system is a hierarchy of multiple oscillators at the organismal, cellular, and molecular levels. At the organismal level, the suprachiasmatic nucleus (SCN) is the apical, central pacemaker that integrates light information and ultimately regulates the rhythms of gene expression, physiology and behavior. At the cellular level, the SCN consists of multiple oscillatory neurons that are coupled into a circadian unit [13, 14]. Overall, biological rhythms and biological clock genes are thus regulated by a complex network of interactions. Synchronization of the biological clock is calibrated by intercellular coupling following signaling from the central pacemaker, which involves neuronal electrical activity, modulation of activators, synaptic transmission, and transmission of information between the SCN and other brain regions and/or peripheral nerves. Knockout of *Bmal1* can abolish circadian rhythms in behavior, blood pressure, and heart rate [11, 12], although this effect is not necessarily observed in a small number of tissues such as fibroblasts [15].

In terms of gross phenotype, mice lacking *Bmal1* display decreased body size and weight [16, 17], as well as abnormal knee joint morphology and calcified tendons [18], indicating that the growth and development of these animals can be greatly affected by *Bmal1*. In addition to impaired growth, *Bmal1* global knockout also leads to significantly lower survival rates in mice [17] and display several signs of premature aging, including sarcopenia, cataracts, subcutaneous fat loss, and organ atrophy [16]. The mechanisms responsible for *Bmal1* deficiency-related aging may involve mammalian target of rapamycin (mTOR) signaling, sirtuins, or nicotinamide adenine dinucleotide (NAD^+^) [19–21]. *Bmal1* is gradually depleted from the nucleus during cellular senescence in human and cynomolgus monkeys. In addition, *Bmal1* has been shown to function in maintaining genomic stability, inhibiting LINE1 transposase activation and antagonizing cellular senescence, which cumulatively suggest that *Bmal1* may inactivate LINE1 that drives aging in primate cells [22]. Furthermore, *Bmal1* knockout was found to induce ovarian dysplasia, significantly reduce follicle and corpora lutea counts, and impair steroid production in female mice. In *Bmal1* knockout male mice, testes, seminal vesicles, and seminiferous tubules are generally reduced in diameter [23–25]. These results suggest a key role for *Bmal1* in reproductive endocrinology and fertility, although we lack an understanding of the underlying mechanisms.

Inflammatory and intracellular immune dysfunction are also strongly associated with defects in *Bmal1*. Knockout of *Bmal1* leads to increased accumulation of reactive oxygen species in macrophages and promotes the accumulation of the hypoxia-responsive protein, HIF-1α, which affects glucose absorption and glycolytic processes, ultimately stimulating pro-inflammatory cytokine IL-1β production [26–28]. Additionally, *Bmal1* can decrease transcription of chemokine ligand 2 to attenuate the number of Ly6C^hi^ monocytes and inflammation [29]. These above findings show that *Bmal1* is an important mediator linking the biological clock with the immune system by limiting inflammatory response. *Bmal1* function is also reportedly relevant to hyperglycemia and hypoinsulinemia, most likely through (1) transcriptional regulation of cAMP-responsive element-binding protein H and apolipoprotein AIV to control larger lipoprotein production [30], (2) regulation of β-cell development and function [31, 32], and (3) regulatory contributions to maintaining metabolic homeostasis to ensure normal mitochondrial function [33, 34].

The relationship between *Bmal1* and tumors is complicate, and its effects may be bidirectional. For example, it has been demonstrated to inhibit cell growth in some cancers, such as neuroblastoma [35], tongue squamous cell carcinoma [36], spontaneous hepatocellular carcinoma [37] and lung tumors [38]. However, *Bmal1* has also been reported as an oncogene [39, 40], such as in acute myeloid leukemia models, where it was shown to be essential for the growth of leukemia stem cell (leukemia stem cell are responsible for disease development and spread). Moreover, disruption of *Bmal1* expression results in anti-leukemic effects [39]. It should be noted that the positive effects of *Bmal1* on tumor growth are very closely related to its function in the regulation of metabolism. In conjunction with other clock genes, *Bmal1* is necessary for metabolic processes in cells by controlling how nutrients and metabolites are utilized in a time-specific manner to support cell proliferation and biomass production [41]. This collective evidence suggests the possibility that *Bmal1* could serve as a potential therapeutic target for tumors.

**Fig. 1:**
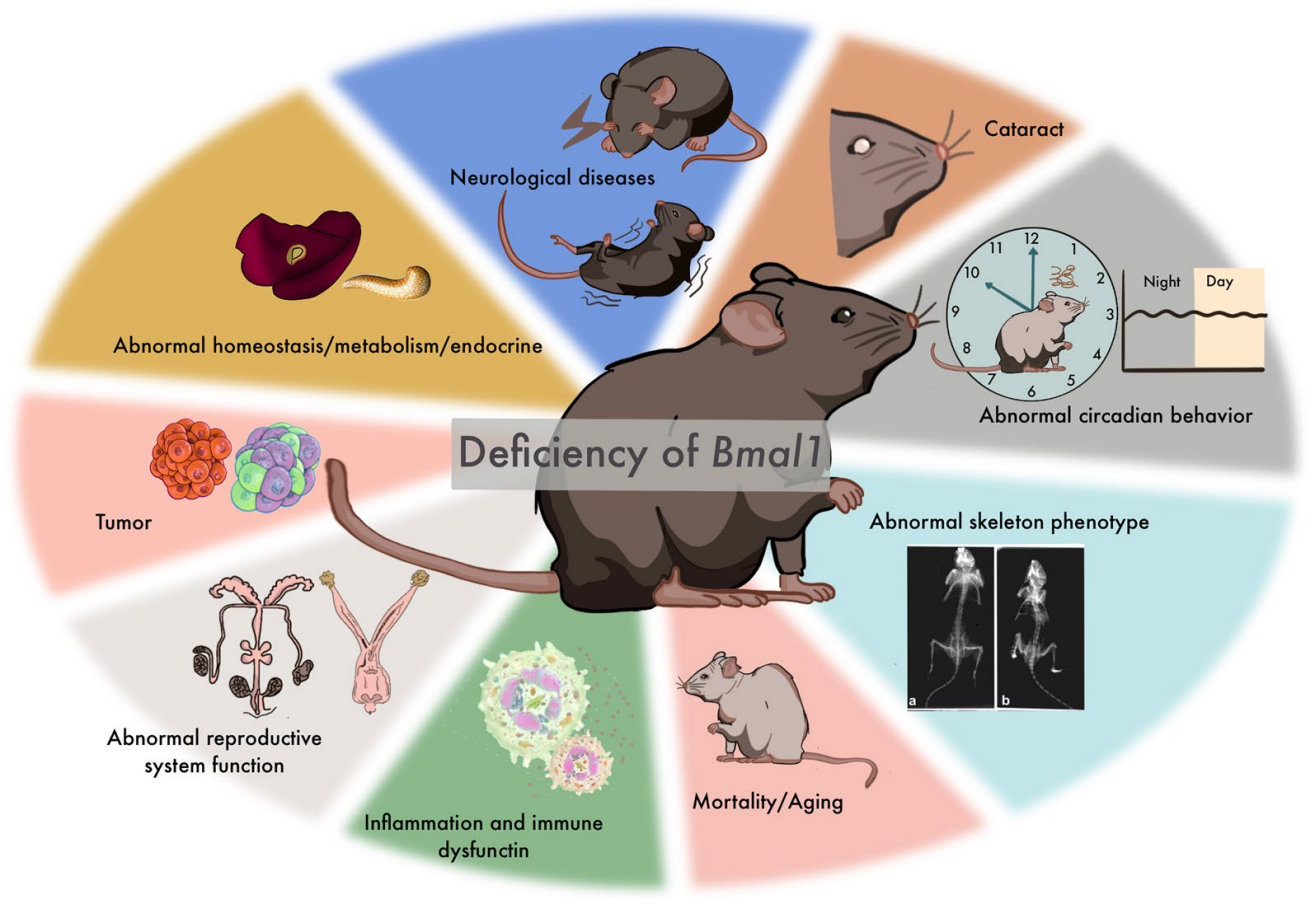
A brief overview of the basic functions of *Bmal1. Bmal1* has important roles in circadian rhythm, vision/image processing, the neurological basis of behaviors, endocrine/exocrine glands, liver/biliary system, homeostasis/metabolism, oncogenesis and tumorprogression, respiratory system, immune system, growth and aging, skeletal system development. Radiographs are from [18]

In addition to these functions, a growing body of evidence supports an important role of *Bmal1* in neurological disorders, especially psychiatric disorders (e.g., depression, schizophrenia) [3, 42] and neurodegenerative pathologies (e.g., Parkinson's syndrome, Alzheimer's disease, etc.) [4, 5, 7]. However, the mechanisms involved are remarkably broad and complex. A brief overview of the basic functions of *Bmal1* is shown in Fig. 1, and a comprehensive perspective of *Bmal1* in neural function is provided in sections below.

## *Bmal1*-mediated neuronal activity and neural circuits

**Fig. 2:**
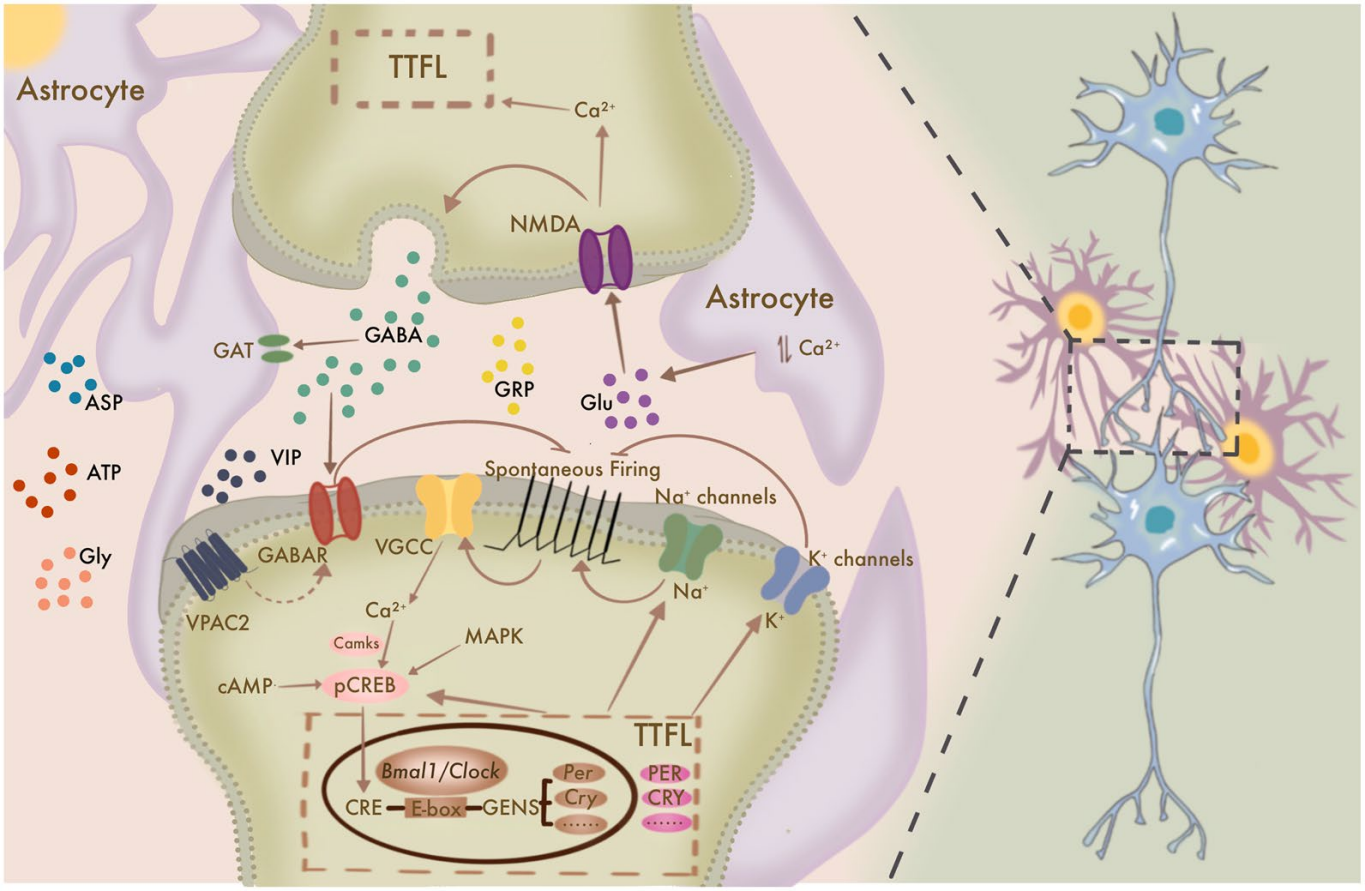
*Bmal1*-mediated neuronal activity and involved neural circuits. A simplified overview of the relationship between the TTFL and neuronal activity. SCN neuronal activity is controlled by a combination of ion channels and signaling pathways. SCN neurons communicate through synapses, various activation factors, and possibly gap junctions to produce rhythms. Astrocyte regulation of rhythms relies on the regulation of glial transmitters such as Glu, ATP, ASP, and Gly. The regulation of Glu release is closely related to oscillations of the GABAergic network. TTFL: Transcription/Translation Feedback Loop; Glu: glutamate; VIP: Vasoactive intestinal polypeptide; GRP: Gastrin Releasing Peptide; AVP: Arginine Vasopressin; GABA: γ-aminobutyric acid; ASP: Aspartic Acid; Gly: Glycine; VPAC2: Vasoactive Intestinal Peptide Receptor 2; VGCC: Voltage-Gated Calcium Channel; NMDAR: *N*-methyl-d-aspartate receptor; GABAR: γ-aminobutyric acid receptor; GAT: GABA transporter

BMAL1 is a major positive feedback regulator of the biological clock, so current research on its relationship with neuronal activity has focused on the SCN. The SCN consists of multiple neuron types including vasoactive intestinal polypeptide (VIP) and Arginine Vasopressin (AVP) positive neurons [43, 44]. Indeed, almost every neuron in the SCN synthesizes γ-aminobutyric acid (GABA), and GABA signaling plays a dominant role in SCN neuronal activity [45–48]. Overall, the neuronal activity and transmission between SCN neurons, mediated by TTFL, are relevant to the spontaneous firing rate (SFR) of SCN neurons and intracellular calcium concentration [49] (Fig. 2).

### Electrical activity and activator signaling pathways in SCN neurons

SCN neuronal activity is biologically rhythmic, with the rhythm generated by a combination of ion channels and signaling pathways. First, cyclic changes in the physiological activity of central pacemaker cells occurring over a 24-h period have been observed in both mammals and drosophila. At night, the SFR and input resistance of SCN neurons are lower than during the day in rodents, whereas depolarization of the resting membrane potential is more pronounced in the daytime phase than at night [50–55]. In the morning, sodium conductance through NA/NA Leak Channel Non-Selective Protein ion channels depolarizes these neurons. Currents are driven by the rhythmic expression of nematode cation channel localization factor-1, which links the molecular clock to ion channel function. At night, basal potassium currents peak, silencing the clock neurons [56]. Calcium-activated potassium channels (BK channels) are inactivated through their N-type β2 subunits, and inactivation of BK currents during the day reduces their steady-state current levels. At night, the inactivation decreases, thereby increasing the BK current. It is reasonable to speculate that the biological clock may regulate circadian changes in cellular excitability by inactivating gating channels [57]. In addition, inhibiting potassium channel (Kv4.1, Kv4.2) gene expression also leads to an increase SFR in SCN and thus a shorter rhythm cycle [58, 59]. In addition to the role of ion channels, longer day length can alter the expression pattern of chloride transporters. Intracellular chloride accumulation leads to greater production of excitatory GABA synaptic inputs by modulating the strength and polarity of the ionotropic γ-aminobutyric acid receptor (GABA_A_R)-mediated synaptic inputs. In contrast, blocking either GABA_A_R signaling, or chloride transporter activity disrupts changes in the phase and cycle induced by light stimuli [60].

Dynamic fluctuations in calcium ions (Ca^2+^) also play a key role in the regulation of biological clock oscillations, especially biological clock gene transcription [61]. Individual neurons in cultured SCN sections exhibit strong circadian fluctuations in response to intracellular Ca^2+^ concentration [62], and Ca^2+^ influx can eliminate the rhythmic expression of biological clock genes. These phenomena suggest that diurnal variation in membrane potential triggered by the cyclic transmembrane influx of Ca^2+^ has an important role in the rhythmic expression of clock genes [63]. In addition, CaMKII (calmodulin-dependent protein kinase II) also participates in synchronization between individual neuronal clocks. For instance, in Rat-1 cells expressing a *Bmal1*-luc reporter exposed to 20 µM KN93 (CaMKII inhibitor), the bioluminescence rhythm is substantially attenuated. Knockdown of CamkIIγ and CamkIIδ by siRNA can also significantly attenuate the amplitude of the *Bmal1*-luc rhythm in NIH3T3 fibroblasts. CaMKII-mediated phosphorylation of CLOCK (i.e., *Bmal1* is not phosphorylated) facilitates its interaction with *Bmal1* and enhances E-box-dependent gene expression [64]. The peak in *Bmal1* transcription occurs before the highest level of action potential, while neuronal activity and *Bmal1*-driven transcription of the biological clock concurrently increase at the beginning of each daily cycle [14].

Apart from Ca^2+^ and CaMKII, the cyclic adenosine monophosphate (cAMP) signaling pathway is also an important pathway involved in coupling membrane potential and clock gene expression. Several studies have demonstrated that cAMP levels are rhythmic in the SCN. The cAMP peak occurs during the day in the SCN, prior to the rhythmic peak of the neuronal activity. Transcriptional activity of the cAMP response element is also strongly rhythmic in the SCN [65–67]. Casein kinase and signaling by RAS-dependent mitogen-activated protein kinases (MAPKs) is also relevant to rhythmicity. Protein kinase C (PKC) and receptor for activated C kinase-1 (RACK 1) have also been identified as components of the biological clock. These Ca^2+^-sensitive signal molecules are recruited to the BMAL1 complex in the nucleus. Overexpression or deletion of RACK1 or PKC may affect the suppression of CLOCK-BMAL1 transcriptional activity and circadian period [68]. Thus, these signaling pathways can also act as 'cytoplasmic' oscillators. In conclusion, neuronal electrical activity, intracellular activator signaling pathways, and the transcription of biological clock genes, such as *Bmal1* are closely related, and they together mediate biological clock rhythms in the SCN. However, whether these interactions also occur in cells outside of the SCN has not yet been reported.

### Inter-neuronal oscillation and neural circuitry

Communication between neurons also follows a biological rhythm. Parvalbumin (PV)-positive neuron-specific deletion of *Bmal1* results in a reduced expression of PV and decreased visual acuity in the visual cortex, whereas *Bmal1* knockout in forebrain pyramidal neurons of TLCN-Cre mice does not, suggesting that *Bmal1* plays an important role in the functional maturation of the PV circuit [69]. In addition, *Bmal1* knockout in astrocytes leads to impaired circadian motor behavior, cognition and prolongs the circadian cycle of SCN clock gene expression, suggesting that circadian rhythms in SCN astrocytes regulate the daily rhythms of the SCN and behavior, like rhythmic activity in SCN neurons. The rhythmic oscillations of the SCN are associated with inter-neuronal transmission, whereas astrocytes are associated with maintenance of the rhythmic cycle [48, 70]. Studies have shown that conditional knockout of *Bmal1* in astrocytes does not completely disrupt the rhythm of SCN clock gene expression but can delay the cycle by 30 min [70]. Similar findings were obtained in mice with astrocyte-specific knockout of other biological clock genes [71]. It is also noteworthy that *Bmal1*-deficient mice exhibit impaired formation of actin stress fibers in astrocytes, leading to morphological changes that can negatively impact synaptic function [72].

In order to produce coherent rhythms, SCN neurons communicate through synapses [73], various active factors [74, 75], and possibly gap junctions [76]. Although individual SCN neurons can act as independent circadian oscillators [13], SCN network connections contribute to enhancing overall cellular rhythmicity [77]. In SCN neurons, a variety of cellular processes follow circadian rhythms, including clock gene expression, Ca^2+^ flux, neuronal firing rates, and neuropeptide release. Furthermore, coupling between SCN neurons requires the involvement of a variety of molecules, including GABA, VIP, and Gastrin Releasing Peptide [78]. This functional coupling is not restricted to neurons, astrocytes also play an important role in neuron-neuron coupling. Synapses in the SCN are "tripartite", consisting of presynaptic axon terminals, postsynaptic membranes, and astrocytes containing GABA transporters [48, 79]. The regulation of rhythm by astrocytes relies on control of glial transmitters such as glutamate (Glu), Adenosine Triphosphate (ATP), Aspartic Acid (ASP), and Glycine (Gly) [80–83]. The coordination of Glu release is closely related to oscillations in GABAergic network. By using microdialysis, rhythmic changes in GABA were detected in the SCN, which functions as the central pacemaker, as well as in other brain regions [84]. Glu can also transmit retinal light information to the SCN via retinal hypothalamic projections [85, 86]. At night, active astrocytes release Glu, which activates the presynaptic NR2C subunit-containing *N*-methyl-d-aspartic acid receptor (NMDAR). In turn, NMDAR stimulates the release of GABA, consequently reducing SCN neuronal activity at night [71], and also triggering periodic oscillations in intracellular Ca^2+^ levels [87]. Investigations of the transcriptional regulatory mechanisms for this oscillation showed that phosphorylation of serine residues in BMAL1 increased in response to Glu stimulation and BMAL1 protein level [88]. Depletion of VIP or VPAC2 knockout both result in the loss of rhythm in SCN brain slices [89], while incubating these SCN slices with wild-type murine SCN brain slices can rescue rhythm in VIP-/VPAC2-deficient SCN tissue [75]. GABA signaling forms connections among neurons in the SCN and helps maintain rhythmic oscillations [90], although antagonizing GABA signaling in normal SCN brain slices does not disrupt the rhythm, and only in the absence of VIP signaling accelerate the loss of rhythm. This finding suggests that VIP signaling may facilitate GABA signaling to antagonize the rhythm. Furthermore, the mechanisms of this GABA-VIP signaling pathway may also play a role in the regulation of intracellular signaling feedback loops by cAMP and Ca^2+^ [45]. These collective findings thus indicate that the maintenance of rhythm is determined by a balance between VIP and GABA signaling. Overall, oscillation and the neural circuit responsible for rhythm is an extremely complex network. While studies examining the networks between SCN neurons and other cells remain limited, further investigation is also needed to determine the specific influence on transmission and mechanisms through which *Bmal1* participates in other neural circuits.

## Expression of *Bmal1* in different cell types in the brain

**Fig. 3:**
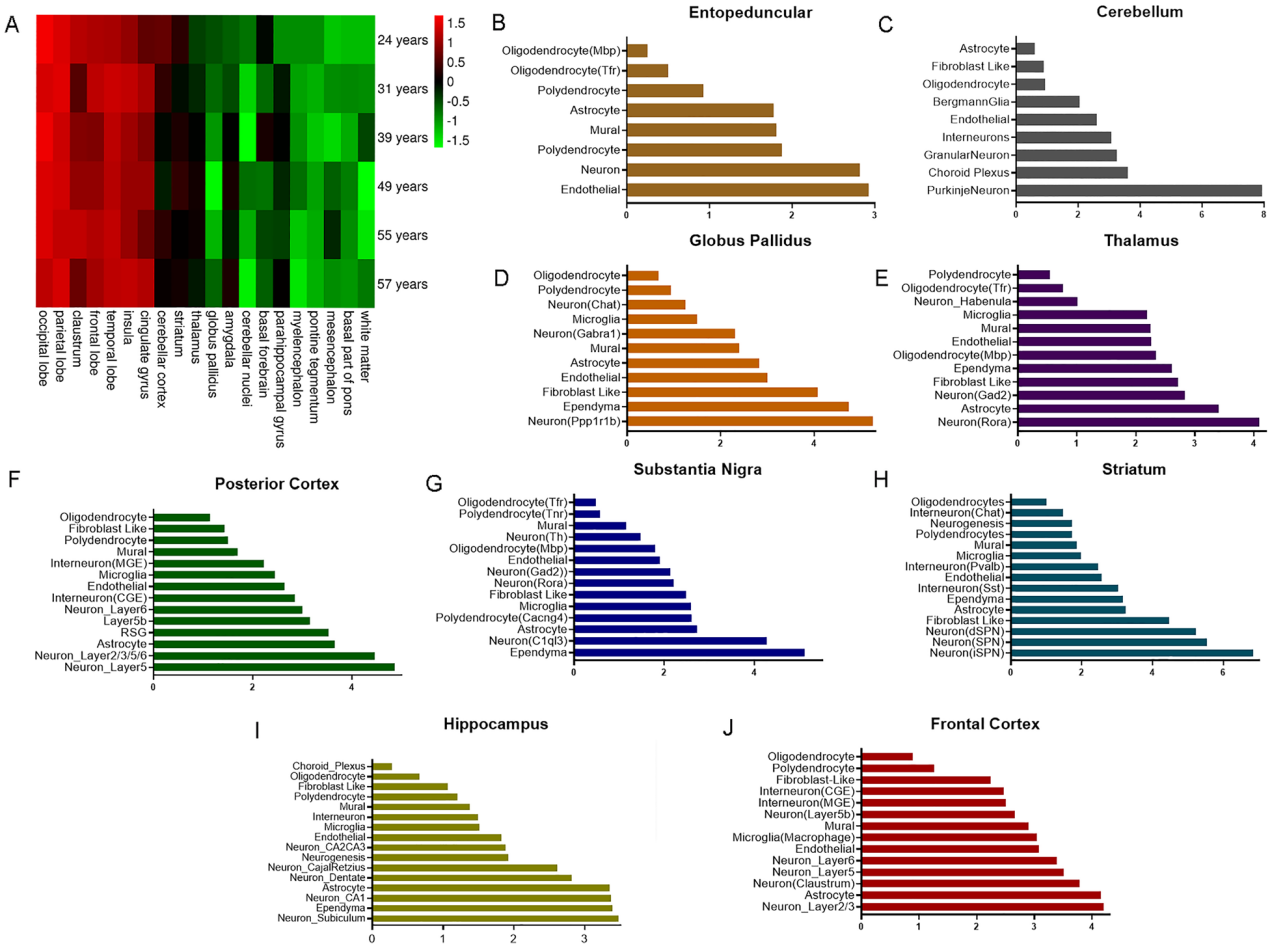
Expression of *Bmal1* on different cell types in the brain. **A** Distribution of *Bmal1* in different regions from six normal human samples (aged 24–57), with green and red in the heatmap indicating low and high expression, respectively. The data were obtained from https://portal.brain-map.org/. **B**–**J** Expression of *Bmal1* in different brain regions of mice. Horizontal axis represents transcripts per 100,000 in each cluster; ordinates represent subsets of cells identified with different markers, with special markers indicated in parentheses. The data were obtained from DropViz website [142]

Based on genomic data from the Allen Institute for Brain Science (ALLEN BRAIN MAP) [91]. *Bmal1* expression patterns in the human brain are similar at different age stage. Specifically, *Bmal1* is more abundantly expressed in brain regions such as the parietal/occipital/frontal/temporal lobes, nucleus accumbens, to a lesser extent in the thalamus, cerebellar cortex, striatum, with relatively low expression in the mesencephalon, cerebellum, corpus callosum and other white matter regions (Fig. 3A). By contrast, *Bmal1* is most abundantly expressed in the isocortex, thalamus and cerebellum in mice, followed by the cortical subplate and hippocampal formation.

During the perinatal period in mice, *Bmal1* was found to be enriched in the cerebral cortex, peaking on postnatal day 3. In utero electroporation combined with RNAi interference experiments in mice revealed that *Bmal1* knockdown in neurons delays their radial migration in the embryonic cortex. Furthermore, reduced *Bmal1* expression throughout the brain disrupts axonal projections from the corpus callosum to the lateral cerebral hemisphere ipsilaterally [92]. A variety of factors, including Glu, Ca^2+^, cyclic AMP-dependent protein kinase (PKA), and diacylglycerol-dependent protein kinase are involved in coordinating *Bmal1* transcription and translation during development [9, 88, 93].

Single-cell RNA sequencing (scRNA-seq) analysis in adult mouse brains [94] revealed abundant *Bmal1* expression in both neurons and glial cells, also with especially high transcript levels in Purkinje cells. *Bmal1* expression varies across different brain regions but with no difference between excitatory and inhibitory neurons (Fig. 3B–J. See the original article for the distribution in specific cell subpopulations). Among specific neurons in different brain regions, scRNA-seq showed that PV-positive interneurons (GAD1/2^+^, PV^+^) in prefrontal cortex (PFC) has highest *Bmal1* expression, twice more than that in excitatory neurons (SLC17A^+^), SST-positive interneurons (GAD1/2^+^, SST^+^) and astrocytes (Gja1^+^). In several brain regions, higher *Bmal1* expression is a feature of inhibitory neurons, such as inhibitory neurons (GAD1/2^+^) and fibroblasts in the posterior cortex, GAD1/2^+^ inhibitory neurons in the striatum, GAD1/2^+^ inhibitory neurons and mural cells in the cerebellum. Whereas in the hippocampus, *Bmal1* expression is higher in excitatory neurons (SLC17A^+^) and astrocytes (Gja1^+^) than inhibitory neurons, while the thalamus has *Bmal1* expressed equally in different cell types.

The detection of *Bmal1* in rat brain by using two neuropeptides (substance P and enkephalin) co-expressed with *Bmal1* and the other clock gene, *Per2,* found *Bmal1* in almost all neurons (~ 90%) in the forebrain (dorsal striatum, nucleus ambiguus, amygdala, and terminal cortex), while *Per2* is expressed in a slightly lower proportion of neurons. In the olfactory bulb, *Bmal1* and *Per2* are expressed only in a smaller proportion of cells [95]. Overall, *Bmal1* is widely expressed in the mammalian brain and is notably abundant in both neurons and glial cells, which likely participates in neuronal development.

## *Bmal1* in neurological diseases

Several studies have found associations between clock genes and neurological diseases. For instance, genes with rhythmic oscillations in their expression, like *Bmal1*, exhibit altered peak timing and phasing in depressed patients [96]. A similar pattern was found in the pineal gland and cingulate cortex of Alzheimer's disease (AD) patients and in leukocytes of Parkinson's disease (PD) patients [4, 97, 98]. Similarly, a reduction in the amplitude of expression rhythms of genes such as *Bmal1* was detected in the saliva of patients with bipolar disorder [99]. Single nucleotide polymorphism (SNP) analysis found polymorphisms in *Bmal1* and other genes that were potentially associated with increased risk of seasonal affective disorder and AD [100–102], while genome-wide association studies revealed a large overlap (> 80%) in the genetic factors involved in bipolar disorder, depression and schizophrenia, including hundreds of significant genetic loci [103], several of which were related to clock genes. A report on SNP markers by the Psychiatric Genomics Consortium (Bipolar Disorder and Schizophrenia Working Group) stated that a SNP in *Bmal1* could help to differentiate genetic risk for bipolar disorder and schizophrenia [104]. This SNP in *Bmal1* was also correlated with morbidity in bipolar disorder and schizophrenia [3, 105, 106].

Aligning well with these above findings, epigenetic mechanisms are known to be related to the regulation of the circadian clock. Aberrant DNA methylation of *Bmal1* was observed in bipolar disorder and AD patients [107, 108]. In addition, the first primate model of deficiency for a core rhythm gene was generated by CRISPR/Cas9-mediated knockout of *Bmal1* in macaque, which resulted in schizophrenia-like symptoms, further supporting a possible role of *Bmal1* in neurological disorders [42]. In humans, robust evidence indicates that chronotherapy is highly effective for treating mood disorders [109]. For example, agomelatine was recently developed as a new antidepressant targeting the biological clock [110]. Activation of *Bmal1* is also a biological target for lithium in the treatment of bipolar disorder [111]. These diverse lines of evidence suggest that *Bmal1* may play a causative role in mental disorders, some of which phenotypically resemble neurological disorders.

*Bmal1* has also been observed to influence different interactions responsible for the onset of neurological disease in animals. For instance, autistic-like behavior has been linked to deficiency of *Bmal1*, with hyperactivation of mammalian target of rapamycin complex 1 (mTORC1) signaling implicated as a likely important contributing pathway [10, 112]. In chronic unpredictable mild stress model rats, clock gene expression in brain subregions hippocampus and nucleus ambiguous, as well as liver, are altered following stress induction, with *Bmal1* and *Per2* levels showing particularly high fluctuations in response to stress [113]. Rats exposed to forced activity (simulating night-shift work), showed no significant changes in their clock-related genes expression in hippocampus, but the phosphorylation of BMAL1 and its regulator S6 kinase beta-1 was significantly reduced in PFC. Thus, simulating night-shift work rats have a disruption in the post-transcriptional regulatory pathway controlling clock genes mRNA translation in PFC, and this disruption may also be associated with impaired arousal during night work [114].

Circadian rhythm plays an important role in immune function, and its disruption has been linked to the etiology of depression. Evidence suggests that chemokines, the production of which are controlled by Clock, contribute to neuroinflammation-induced depression, therefore implying that clock genes may also serve as regulators of neuroinflammation [115]. Dopamine (DA) D2 receptor-mediated signaling can enhance the CLOCK:BMAL1 complex capacity for transcriptional activation of its targets [116], while AHI1 (frequently associated with abnormal neurodevelopment and mental disturbance) is known to bind RORα and repress BMAL1 expression, subsequently inhibiting Rev-Erbα expression and increasing tyrosine hydroxylase expression [117]. These studies provide an intriguing connection between abnormalities in circadian rhythm in mental disorders and the dopaminergic hypothesis. Overall, *Bmal1* may be responsible for the onset and progression of several psychiatric disorders through multiple pathways.

Alternatively, *Bmal1* regulates neuroinflammation in the brain to maintain functionality of the DA signaling pathway, whereas disruption of this balance has been proposed as causative factor in the onset of PD [118]. In transgenic dominant negative *Bmal1* mice, hippocampal regulation of memory retrieval via DA and PKA-induced GluA1 phosphorylation [9], suggesting that *Bmal1* could be relevant to neurodegenerative pathologies through DA signaling pathway. Apart from DA signaling, *Bmal1* deletion in mice was shown to result in activation of astrocyte proliferation [119], causing the development of abnormal pathological phenotypes such as memory impairment and hyperactivity [120]. By contrast, elevation of *Bmal1* expression leads to impaired astrocyte function via inhibition of aerobic glycolysis [121]. Additionally, methylation of CpG sites in the *Bmal1* promoter can lead to its epigenetic silencing, which has been linked with the pathological progression of AD [122, 123]. Post-translationally, accelerated BMAL1 degradation also leads to circadian rhythm disruption in AD mouse model [124]. Currently, considerable research efforts are dedicated to defining the role of *Bmal1* in AD and its potential as a therapeutic target, which has been well-reviewed by Ashish Sharma and colleagues [125].

## Pathological phenotypes arising from *Bmal1* deletion

Major advances in gene editing have facilitated the establishment of *Bmal1* knockout animal models to enable deeper investigation of its neurobiological functions. In various *Bmal1* knockout mice model, its deletion triggered not only circadian rhythm-related disorders, but also psychiatric disorders, memory impairment, and other neurological disorders with different disease phenotypes associated with specific brain regions and/or cell subpopulations subjected to conditional deletion (see Table 1 for details). In addition to biological clock disruption, consequently altering behavioral rhythms and biological clock gene expression, global *Bmal1* knockout also leads to degeneration of synaptic terminals, impaired functional connectivity in the cortex, oxidative damage to neurons, and impaired expression of several redox defense genes [120]. Behaviorally, these *Bmal1* knockout mice display hyperactivity, deficiency in short- and long-term memory formation in novel environments [126], impairment of social behaviors and increased stereotyped behavior [10, 112]. In mice with *Bmal1* knockdown by intra-cerebroventricular injection of siRNA, both activity and waking time are reduced, while sleep in the dark phase and immobilization in tail suspension tests increased [8]. Since SCN serves as the master clock brain region coordinating biological clock, specific labeling or pathological changes following chemogenetic interference with *Bmal1* in this area can be highly informative of its function, as reported in numerous studies. Mice with *Bma1* knockdown by viral injection in the SCN exhibited depressive- and anxiety-like behavioral changes, like slower escape from stress in learned helplessness, increased immobility time in tail suspension tests, and less time spent in the bright box in light–dark transition test, as well as increased body weight and an overall decrease in corticosterone release with an abnormal release rhythm [127]. Synaptotagmin10 (Syt10) is highly expressed in the SCN but is expressed at relatively low levels in other regions, make it as a perfect marker for SCN cells. *Bmal1* expression was reduced by 65% in heterozygous Syt10-Cre mice (Syt10-Cre^+/−^; *Bmal1*^loxp/loxp^), which did not result in circadian arrhythmia, whereas *Bmal1* transcript levels decreased by 83% in homozygous Syt10-Cre mice (Syt10-Cre^+/+^; *Bmal1*^loxp/loxp^) that was accompanied by arrhythmia [128]. In addition, by crossing Neuromedin S-Cre mice (specific labelling of SCN neurons) with Bmal1^loxp/loxp^, mice showed a ~ 32% reduction in *Bmal1* mRNA levels in the SCN and resulted in disturbance in circadian rhythm-associated behavior [129].

**Table 1:** Recent advances in abnormal *Bmal1* expression and phenotype research through gene editing

Target Subcellular units	Knockout Strategies	Rhythmic phenotype		Behavioural phenotype		Other phenotypes		References
Global	Conventional knockout		/		/	+	Astrogliosis	[120]
						+	Synaptic terminals	
						+	fcOIS	
Global	Conventional knockout	+	Rhythmic expression of clock genes in brain	+	Novelty-induced hyperactivity	+	Reactive oxygen species homeostasis	[126]
Global	Conventional knockout	+	Expression of clock gene in microglia		/	+	Expression of pro-inflammatory cytokines, antioxidative and anti-inflammatory factors	[143]
Global	Conventional knockout		/	+	Social behaviours	+	Excitatory synaptic transmission	[10]
				+	Stereotyped and repetitive behaviors	+	Spontaneous firing	
				+	Motor coordination	+	mTOR signaling	
				+	Anxiety-like behavior	+	Density and morphology of dendritic	
				−	Depressive-like behavior		Spines in Purkinje cell	
Global	Conventional Knockout		/	+	Working memory		/	[130]
				+	Hippocampal-dependent memory			
Global	Conventional knockout		/	+	Vocalizations during maternal separation	+	mTOR signalling	[112]
	(heterozygote)			+	Social behaviours			
				+	Tereotyped and repetitive behaviors			
				+	Anxiety-like behavior			
				+	Motor coordination			
				−	Novel object recognition memory			
Most neurons, astrocytes	Nestin Cre	−	Behaviour rhythm	−	Response to novel environments	+	Astrogliosis	[120]
and oligodendrocytes	× *Bmal1* ^f/f^					+	Microglia activation	
(except microglia)		+	Rhythmic expression of clock genes in cortex			+	Expression of Nqo1 and Aldh2 (related to oxidative stress regulation) in cortexs	
Whole Brain	siRNA ICV injection	−	Behaviour rhythm	+	Depressive-like behavior	−	Orexin A, CRH, GABA levels	[8]
		+	PSG: sleep/wake changes					
SCN	Short hairpin RNA	+	Behaviour rhythm	+	Learned helplessness paradigm	−	Corticosterone levels	[127]
	Injection into SCN	+	Rhythmic expression of clock gene	+	Depressive-like behavior			
				+	Anxiety-like Behavior			
				+	Weight			
Most cells in SCN	Synaptotagmin10 Cre	+	Behaviour rhythm		/		/	[128]
	× *Bmal1* ^f/f^							
SCN	Neuromedin s Cre	+	Behaviour rhythm		/		/	[129]
	× *Bmal1* ^f/f^							
Excitatory neurons	CaMKII-Cre		/	+	Hippocampal-dependent memory		/	[130]
in forebrain	× *Bmal1* ^f/f^							
Excitatory neurons	CaMKII-Cre	+	Behaviour rhythm	+	Learning and memory		/	[131]
in forebrain	× *Bmal1* ^f/f^			−	Depressive-like behavior			
				−	Anxiety-like Behavior			
Forebrain	Inhibition of BMAL1 function (dnBMAL1)	−	Behaviour rhythm	+	Memory retrieval	+	DA-cAMP signalling	[9]
	× CaMKII-tTA			−	Anxiety-like Behavior	+	Phosphorylation of GluA1 S845	
Neurons in forebrain	Camk2a:: iCre BAC	+	Behaviour rhythm		/		/	[132]
and SCN	× *Bmal1* ^f/f^	+	Rhythmic expression of clock gene					
GABAergic	Vgat-Cre	+	Behaviour rhythm		/		/	[133]
and glycinergic neurons	× *Bmal1* ^f/f^							
Astrocytes	Glast -CreER	+	Behaviour rhythm	+	Short- and long-term memory	+	Expression of VIP in the SCN	[48]
	× *Bmal1* ^f/f^	+	Rhythmic expression of clock gene in the cortex and hippocampus			+	Expression of GABA transporters in Astrocytes	
						+	GABA levels	
Astrocytes	Glast -CreER	+	Rhythmic expression of clock gene in hypothalamic		/	+	Energy balance	[138]
	× *Bmal1* ^f/f^					+	Glucose homeostasis	
						+	Lifespan	
						+	Weight	
						+	Astrogliosis in the cortex and hippocampus	
						+	Glu/GABA levels	
Astrocytes in the SCN	*Bmal1* guide RNAs injected into the SCN of Aldh1L1-Cre mice	+	Behaviour rhythm		/		/	[70]
Astrocytes	Aldh1L1-Cre ERT2		/		/	+	Astrogliosis	[119]
	× *Bmal1* ^f/f^					+	Expression of Chi3l1 and Mmp14	
							(Alzheimer’s related genes)	
Astrocytes in the AD model	Aldh1l1-CreERT2		/		/	+	Astrogliosis	[119]
(rapid plaque formation)	× *Bmal1* ^f/f^					−	Fibrillar amyloid plaques	
	× APP/PS1-21					−	Dystrophic neuropil	
						−	Microglia activation	
Astrocytes in the AD model	Aldh1l1-Cre ERT2		/		/	+	Astrogliosis	[119]
(slow plaque formation)	× *Bmal1* ^f/f^					−	Aβ plaque deposition	
	× APP ^NL−G−F/wt^					−	Dystrophic neuropil	
Astrocytes in NAc	AAV8-GFAP-Cre		/	+	Motor response to novelty	+	Ratio of AMPA/NMDA EPSC in MSNs	[139]
	injected into the NAc region of the *Bmal1* ^f/f^ mice			+	Anxiety-like Behavior	+	Expression of glutamate receptors, Gclc, Pgc1α, Ldha, Mct1 and Mct2 (associated with glutathione production, mitochondrial function and lactate synthesis/metabolism)	
						+	Concentrations of glutathione and lactate	
Microglia	Cx3cr1 Cre		/	+	Long-term memory	+	Microglial phagocytosis	[141]
	× *Bmal1* ^f/f^			+	Spatial learning and memory	+	POMC immunoreactive neurons	
						+	Food intake	
						+	Mature dendritic spines	
Purkinje cells	L7-Cre	−	Behaviour rhythm	+	Social behaviours	+	Excitatory and inhibitory synaptic transmission	[10]
	× *Bmal1* ^f/f^			+	Stereotyped and repetitive behaviors	+	Spontaneous firing	
				+	Motor coordination	+	mTOR signalling	
						+	Purkinje cell dendrites	
AVP neurons	AVP-Cre	+	Behaviour rhythm		/		/	[140]
	× *Bmal1* ^f/f^	+	Rhythmic expression of clock gene in SCN					
PV cell	PV-Cre ER		/	+	Visual acuity	+	PV cells in the visual cortex	[69]
	× *Bmal1* ^f/f^							
MSNs in the striatum	Gpr88-Cre	−	Behaviour rhythm	+	Voluntary alcohol intake		/	[134]
	× *Bmal1* ^f/f^							
MSNs in the striatum	Gpr88-Cre	+	Rhythmic expression of clock gene in striatum	+	Anxiety-like Behavior	−	Mitochondrial respiration	[135]
	× *Bmal1* ^f/f^			−	Depressive-like behavior			
				+	Motor coordination			
Neurons in the DG of hippocampus	Syn1-Cre AAV virus injected into the DG of *Bmal1* ^f/f^ mice		/	+	Seizures induced by pilocarpine administration		/	[136]
CRH neurons in PVN	CRH-Cre	+	Rhythmic of calcium activity in CRH neurons of PVN		/		/	[144]
	× *Bmal1* ^f/f^							
		+	Corticosterone release rhythm					
CRH neurons	CRH-Cre	−	Behaviour rhythm		/		/	[137]
	× *Bmal1* ^f/f^	−	EEG and EMG					

fcOIS: optical intrinsic signal functional connectivity imaging; PSG: polysomnographic recording; ICV: intracerebroventricular; EEG: electroencephalogram; EMG: electromyogram; mTORC1: mammalian target of rapamycin complex 1; NAc: nucleus accumbens; EPSC: excitatory postsynaptic currents; MSNs: moderately spiny neurons; POMC: pro-opiomelanocortin; AVP: arginine vasopressin; PV: parvalbumin; PVN: paraventricular nucleus; CRH: corticotropin-releasing hormone; DA: Dopamine. (+) indicates an abnormal phenotype has been observed; (−) indicates no difference was found; / indicates that the article does not contain information about the indicated topic/heading

A growing number of studies have found that *Bmal1* knockdown in different cell types also results in a variety of different pathological states. Using CaMKII: CaMKII-Cre or CaMKII-tTA mice to induce specific deletion of forebrain excitatory neurons while preserving the integrity of *Bmal1* in the SCN revealed significant memory impairment without anxiety or depression-like behavior. And several findings suggested that memory impairment may be related to molecules involved in DA/cAMP signaling in the hippocampus [9, 130, 131]. *Bmal1* knockout in the forebrain and in most SCN cells by crossing CamKIIalpha iCre BAC with *Bmal1* loxp mice resulted in progeny with aberrations in their circadian rhythm-related behaviors, characterized by abolished synchronization between rhythms (although still present) in peripheral tissue with that of the SCN master clock [132], suggesting a functional diversity of *Bmal1* in different cell types.

In Vgat-Cre mice, with GABAergic-specific *Bmal1* knockout leads to behavioral manifestations of circadian rhythm disorders [128, 133]. In mice with striatum-specific knockdown of *Bmal1* in neutrophilic multigrade spiny neurons, mice displayed normal circadian rhythms but voluntary alcohol intake are altered with anxiolytic and antidepressant responses [134, 135]. Moreover, *Bmal1* knockdown in DG neurons of the hippocampus resulted in increased susceptibility to epileptic symptoms induced by trichothecene [136]. Furthermore, *Bmal1* knockout in adrenocorticotropin-releasing hormone (CRH) neurons of the hypothalamic paraventricular nucleus, which has monosynaptic efferent from SCN neurons, induced alterations in the rhythms of neuronal calcium activity as well as corticosterone release. However, in mice with *Bmal1* knockout in CRH neurons across all brain regions, circadian rhythm and sleep electroencephalograms remains intact [137].

In addition to targeting neurons, several studies have examined the effects of *Bmal1* deficiency in other cell populations. In astrocytes, *Bmal1* knockout using the glial Glu and aspartate transporter (Glast-Cre) results in molecular clock impairment in the hypothalamus, and alters circadian motor behavior, cognition and lifespan, affecting metabolic balance and glucose homeostasis. Increased Glu and GABA levels were also observed in hypothalamic of mice with astrocyte hyperplasia. However, modulation of GABA_A_ receptor signaling can fully restore Glu levels, and delay glial hyperplasia and metabolic disorders, ultimately extending lifespan. Suggested that GABA signaling may also regulate neuronal clock activity, potentially promoting metabolic dysfunction and cellular senescence [48, 138]. SCN-specific knockdown of astrocytic *Bmal1*, by using the aldehyde dehydrogenase 1 family member L1 (Aldh1L1)-Cre label astrocytes was shown to prolong the circadian cycle of clock gene expression in SCN, suggesting that astrocytes in the SCN, like SCN neurons, can regulate daily rhythms of gene expression in the SCN and animal behavior [70]. Global, astrocyte-specific knockout of *Bmal1* can promote astrocyte activation [119]. However, *Bmal1* knockout in astrocytes does not affect Aβ plaque burden, dystrophic neurites, or microglial activation in AD model mice [119], thus supporting a relationship between *Bmal1*-mediated astrogliosis and AD.

In addition to master clock brain regions, NAc-specific knockdown of *Bmal1* (*i.e.,* in brain regions related to the reward system) resulted in changes of daytime exploratory drive behaviors, glutamatergic signaling to adjacent medium spiny neurons, and metabolism-related functions (such as lactate and glutathione concentrations), suggesting that *Bmal1* also have effects on the reward system [139]. As AVP has been identified as critical for SCN output, *Bmal1* knockout in AVP-Cre mice did not result in dysrhythmias but instead led to prolonged activity cycles, suggesting an impaired synchronization between SCN neurons [140]. The absence of *Bmal1* in microglia can also lead to varying degrees of memory impairment, although phagocytosis of these cells is increased [141]. *Bmal1* deficiency in Purkinje cells leads to dysmotility and autistic-like behavior, accompanied by deranged inhibitory/excitatory synaptic transmission and reduced spontaneous firing rates [10]. Together, *Bmal1* can function as a biological clock regulator, but its dysfunction can trigger other neurological disorders, both in neurons and glial cells.

In conclusion, evidence from model mice with conditional ablation of *Bmal1* in different brain regions or cell types demonstrates its wide range of physiological roles involving biological clock rhythms, behavior, and even metabolic homeostasis. However, despite this wealth of available evidence, our perspective remains limited regarding the neurological related functions of *Bmal1*, which may be resolved with further investigation of its cell-specific functions. These local or cell type specialized functions also increase the difficulty and complexity of *Bmal1* research, and it remains unclear whether there are other regulatory effects independent of biological clock rhythms. Further division of related studies based on brain region or different cellular subpopulations will help to better define the regulatory role of the *Bmal1* gene in neurological diseases.

## Conclusion

This review delineates the role of *Bmal1* in neural function. Currently, studies in a variety of animal models suggests that *Bmal1* might contribute to the development of neurological disorders, providing a non-trivial body of evidence supporting that changes in *Bmal1* gene-related loci or *Bmal1* expression itself may be associated with various neurological disorders. However, considerable work is still needed to comprehensively depict the mechanisms by which *Bmal1* could mediate the development of neurological disorders. According to our current understanding, *Bmal1* shares a complex relationship with SCN neuronal activity, and its role in circadian oscillatory coupling involves not only different cell types in the SCN, such as neurons and astrocytes, but also several important molecular signals, including GABA, Glu, VIP, and others. In the synchronization and maintenance of rhythm or neural circuitry, these factors function as part of a sophisticated network. Thus, *Bmal1* obviously does not function in isolation, and is central to this wide network controlling overall neuronal activity, coupling between neurons, and positive feedback-based synchronization of rhythmic oscillations in the transcription of other biological clock genes.

## Data Availability

All data are included in the manuscript.

## Abbreviations

TTFL: Transcription/translation feedback loop
SCN: Suprachiasmatic nucleus
mTOR: Mammalian target of rapamycin
VIP: Vasoactive intestinal polypeptide
AVP: Arginine Vasopressin
GABA: γ-Aminobutyric acid
SFR: Spontaneous firing rate
GABA-A: γ-Aminobutyric acid receptor
cAMP: Cyclic adenosine monophosphate
MAPKs: Mitogen-activated protein kinases
PKC: Protein kinase C
RACK 1: Receptor for activated C kinase-1
PV: Parvalbumin
Glu: Glutamate
ATP: Adenosine Triphosphate
ASP: Aspartic Acid
Gly: Glycine
NMDAR: *N*-Methyl-d-aspartic acid receptor
PKA: Protein kinase
scRNA-seq: Single-cell RNA sequencing
PFC: Prefrontal cortex
AD: Alzheimer's disease
PD: Parkinson's disease
SNP: Single nucleotide polymorphism
mTORC1: Mammalian target of rapamycin complex 1
DA: Dopamine
Syt10: Synaptotagmin10
CaMKII: Calmodulin-dependent protein kinase II
CRH: Adrenocorticotropin-releasing hormone
Aldh1L1: Aldehyde dehydrogenase 1 family member L1
GRP: Gastrin Releasing Peptide
ASP: Aspartic Acid
Gly: Glycine
VPAC2: Vasoactive Intestinal Peptide Receptor 2
VGCC: Voltage-Gated Calcium Channel
GAT: GABA transporter
fcOIS: Optical intrinsic signal functional connectivity imaging
PSG: Polysomnographic recording
ICV: Intracerebroventricular
EEG: Electroencephalogram
EMG: Electromyogram
NAc: Nucleus accumbens
EPSC: Excitatory postsynaptic currents
MSNs: Moderately spiny neurons
POMC: Pro-opiomelanocortin
PVN: Paraventricular nucleus
